# A Comparative Study of Non-Viral Gene Delivery Techniques to Human Adipose-Derived Mesenchymal Stem Cell

**DOI:** 10.3390/ijms150915044

**Published:** 2014-08-26

**Authors:** Nur Shuhaidatul Sarmiza Abdul Halim, Kamal Shaik Fakiruddin, Syed Atif Ali, Badrul Hisham Yahaya

**Affiliations:** 1 Regenerative Medicine Cluster, Advanced Medical and Dental Institute (AMDI), Universiti Sains Malaysia, Penang 13200, Malaysia; E-Mail: shuemiza_2003@yahoo.com; 2 Stem Cell Laboratory, Haematology Unit, Cancer Research Centre, Institute for Medical Research (IMR), Jalan Pahang 50588, Malaysia; E-Mail: kamal@imr.gov.my; 3 Cluster for Oncological and Radiological Sciences, Advanced Medical and Dental Institute (AMDI), Universiti Sains Malaysia, Penang 13200, Malaysia; E-Mail: ali2@amdi.usm.edu.my

**Keywords:** mesenchymal stem cell, gene therapy, genetic manipulation, *ANGPT-1* gene, transfection, microporation, proliferation and differentiation

## Abstract

Mesenchymal stem cells (MSCs) hold tremendous potential for therapeutic use in stem cell-based gene therapy. *Ex vivo* genetic modification of MSCs with beneficial genes of interest is a prerequisite for successful use of stem cell-based therapeutic applications. However, genetic manipulation of MSCs is challenging because they are resistant to commonly used methods to introduce exogenous DNA or RNA. Herein we compared the effectiveness of several techniques (classic calcium phosphate precipitation, cationic polymer, and standard electroporation) with that of microporation technology to introduce the plasmid encoding for angiopoietin-1 (ANGPT-1) and enhanced green fluorescent protein (eGFP) into human adipose-derived MSCs (hAD-MSCs). The microporation technique had a higher transfection efficiency, with up to 50% of the viable hAD-MSCs being transfected, compared to the other transfection techniques, for which less than 1% of cells were positive for eGFP expression following transfection. The capability of cells to proliferate and differentiate into three major lineages (chondrocytes, adipocytes, and osteocytes) was found to be independent of the technique used for transfection. These results show that the microporation technique is superior to the others in terms of its ability to transfect hAD-MSCs without affecting their proliferation and differentiation capabilities. Therefore, this study provides a foundation for the selection of techniques when using *ex vivo* gene manipulation for cell-based gene therapy with MSCs as the vehicle for gene delivery.

## 1. Introduction

In recent years, the potential of multipotential adult mesenchymal stem cells (MSCs) for cell-based therapy has received tremendous attention, as transplantation of these cells has proven to be effective at treating a variety of genetic or acquired diseases. This is because MSCs can engraft in various tissue types to differentiate into tissue-specific cells and release trophic factors to induce the tissue’s own endogenous repair [1]. MSCs avoid and/or suppress the immunological responses that cause rejection of most allogeneic cells and tissues, a trait that helps explain how these cells modify the triggering and effector functions of innate and adaptive immunity [2].

Despite the hope that stem cell-based gene therapy will have a positive impact on human health, the use of viral-based vectors to transfer the gene of interest into stem cells remains problematic and controversial [3]. The advantage of using a viral-based vector in gene therapy applications is that it allows for long-term expression of the gene of interest. In contrast, nonintegrating vectors, such as adenoviruses and non-viral gene delivery systems, are preferable for treating non-inherited diseases because expression of the therapeutic gene is transient [4]. Although non-viral methods have lower efficiency compared to viral-based methods, they are safe, noninfectious, and nonimmunogenic, have negligible toxicity, can be produced simply on a large scale, and have the ability to carry larger therapeutic genes [5].

As for translational research of from bench-to-bedside approach in developing therapies for clinical applications, there is increased interest in the development of a safe and efficient non-viral gene delivery system that can overcome the limitations associated with the viral approach. An efficient stem cell-based gene therapy application requires that the modification and transfection strategies not affect the ability of MSCs to proliferate and differentiate. A number of non-viral systems used for gene transfer are currently in use, including the liposome-based method, electroporation, and calcium phosphate techniques [6,7,8]. Among the current non-viral methods, the liposome carriers and the electroporation-based gene transfer techniques are widely used and are thought to be the most efficient for transfecting genes of interest into MSCs [6,7,9,10]. Electroporation, while effective for transfecting genes into stem cells, is rather harsh and leads to excessive cell death [11]. In contrast to the standard electroporation method, microporation is a unique electroporation technology that uses a pipette tip as an electroporation space and a capillary type of electric chamber instead of a cuvette; these modifications reduce the detrimental effects of cuvette-based electroporation gene transfer techniques [4].

In this study, human adipose-derived MSCs (hAD-MSCs) were used to compare the transfection efficiency and toxicity of chemically mediated transfection (classic calcium phosphate precipitation and cationic polymer), the standard electroporation technique, with the microporation technique. The rationale of using hAD-MSCs in this study is because they exhibit some superior properties compared to others adult MSCs. For example, hAD-MSCs expand faster than BM-MSCs when cultured *in vitro*. The present study provides useful insight into effective transfection protocols that do not compromise the stemness, viability, and differentiation capabilities of hAD-MSCs. Ultimately the aim is to express a therapeutic gene *in vivo* in a safe and a transient manner without affecting the proliferation and differentiation capabilities of the recipient cells.

## 2. Results

**Figure 1:**
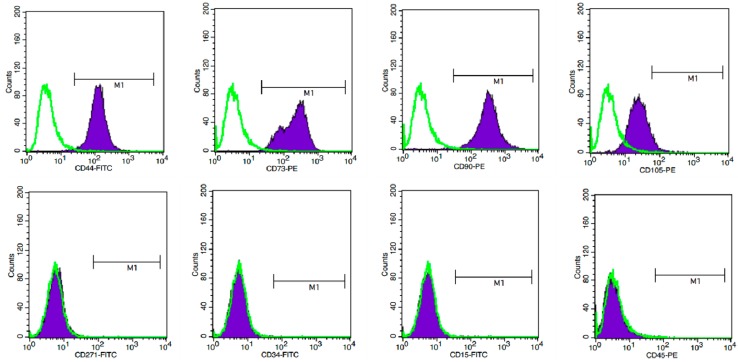
Representative flow cytometric analyses of cultured human adipose-derived mesenchymal stem cells (hAD-MSCs) characterization. Unfilled histograms indicate isotype-matched mouse immunoglobulin G antibody control staining and filled histogram indicate the specific antibody. hAD-MSCs were strongly positive for the CD44, CD73, CD90, and CD105 markers and negative for CD271, CD34, CD15, and CD45. Experiment of each antigen is also indicated as percentage.

In an attempt to study stem cell potentiality, the expression of MSC-related cell surface antigens was evaluated by flow cytometry (Figure 1). hAD-MSCs were strongly positive for the CD44, CD73, CD90 and CD105 markers. However, the hAD-MSCs stained negatively for CD271, CD34, CD15 and CD45.

To establish a reliable transfection protocol for hAD-MSCs, we tested and compared the transfection efficiency of four independent non-viral transfection techniques. Different protocols were optimized by transfecting hAD-MSCs with a plasmid encoding for a reporter gene, *eGFP*, which enabled us to monitor the number of transfected cells to determine the transfection efficiency. For each of the two transfection reagents used in this study (*i.e.*, calcium phosphate precipitation and cationic polymer), the amount of DNA and transfection reagents were optimized following the manufacturer’s instructions, and for both electroporation techniques the various “preset” electroporation programs were optimized. Only the optimal conditions, *i.e.*, in term of transfection efficiency, cell survival, reagent/DNA ratios were compared in this study (supplementary information). The success of the transfection strategies was measured using fluorescence microscopy.

**Figure 2:**
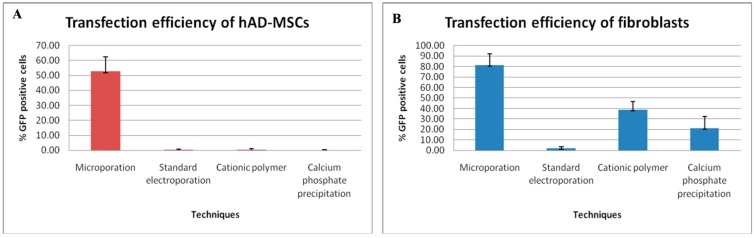
Transfection efficiencies of hAD-MSCs and fibroblasts using four independent techniques. The microporation technique had the highest transfection efficiency as compared to standard electroporation and chemical-based transfection reagents for transfection of (**A**) hAD-MSCs and (**B**) fibroblasts. hAD-MSCs and fibroblasts were transfected with a plasmid encoding for Angiopoietin-1 (ANGPT-1) and the enhanced green fluorescent protein (eGFP) expression cassette using different chemical-based reagents and electroporation protocols after optimization.

A leading electroporation system, the Neon™ Transfection System (Invitrogen-Life Technologies, Grand Island, NY, USA) was used to optimize transfection efficiency and viability of hAD-MSCs and fibroblasts. First we followed the protocol provided by the manufacturer. To further determine the best microporation conditions, equivalent quantities of both cells and plasmid DNA were used to test seven different programs: (P1–P7) in which the pulse magnitude was varied from 1000 to 1600 V and the pulse duration (ms) and frequency (pulse number) were held constant at 20 ms and 1 per transfection, respectively. All of the microporation conditions tested with exception of P1, P2 and P3 led to high transfection efficiency of hAD-MSCs (supplementary information). Transfection efficiency generally increased with increasing voltage, reaching a maximum transfection efficiency of approximately 50% at 1600 V (P7) (supplementary information). However, increasing voltage also induced greater cell death. At voltage pulse greater than 1500 V, cell viability plummeted to 10% or lower. The optimal transfection conditions associated with higher number of cells been transfected with minimal cell death was attained at a pulse magnitude and duration of 1500 V and 20 ms respectively. The efficiency of transfection and the viability were 50% and 78% respectively (Figure 2A and supplementary information). We further observed that cells morphology was retained and that the cells were able to spread following microporation, even at highest voltage tested in this study (data not shown).

**Figure 3:**
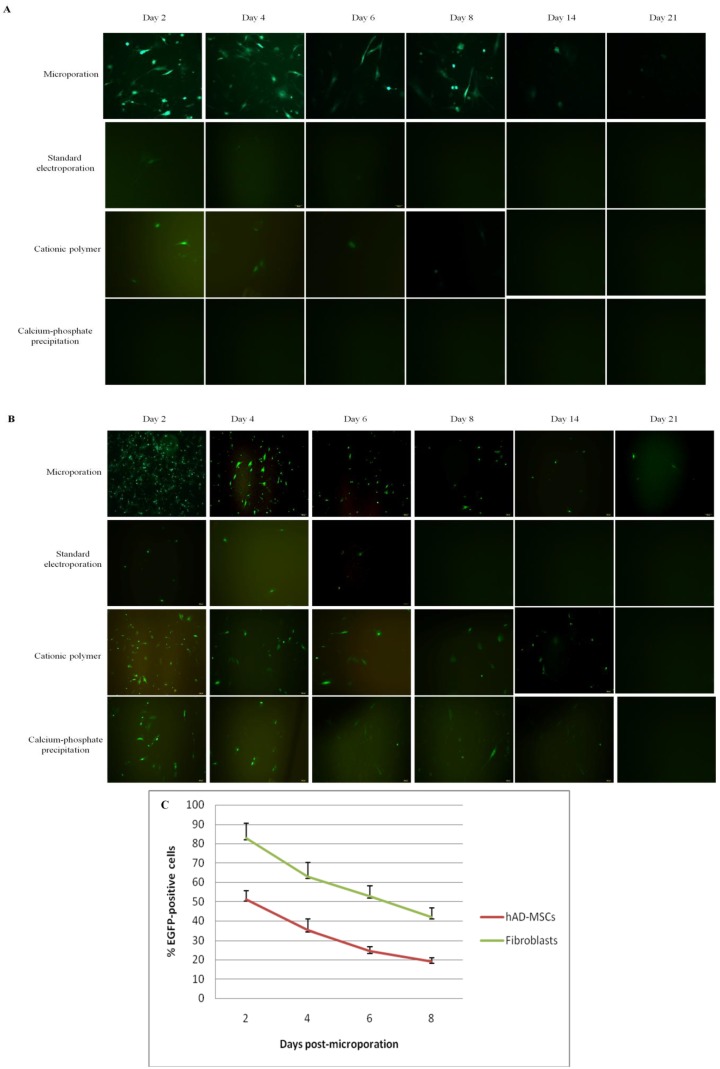
Time-course analysis of eGFP expression level in hAD-MSCs and fibroblasts. Analysis of eGFP expression of (**A**) hAD-MSCs and (**B**) fibroblasts following transfection using the microporation, standard electroporation, cationic polymer, and classical calcium phosphate precipitation techniques over a 21-day period (Magnification: (**A**) 100× and (**B**) 100×); (**C**) Evaluation of eGFP expression in microporated hAD-MSCs and fibroblasts over an 8-day period. The expression of eGFP was detected by fluorescence microscopy at days 2, 4, 6, and 8 following transfection. The number of eGFP-expressing cells began to decline over time.

When using the optimized microporation protocol, the expression of eGFP in hAD-MSCs was as high as 50%. In contrast, no eGFP-positive cells were observed in the hAD-MSC culture following transfection using the calcium-phosphate precipitation method, and only few eGFP-positive cells were observed in the culture when the standard electroporation and cationic polymer techniques were used (Figure 2A and Figure 3A and supplementary information). The number of eGFP-positive cells was measured for up to 21 days, but the number of positive cells was very low by this time point. This observation indicates that expression levels gradually decreased with incubation time (Figure 3A). In the fibroblast cells that were transfected using the microporation technique, P5 generated approximately 80% number of cells that were positive for eGFP with minimal cell death (80% viability) than other pulsing programs (supplementary information); the values were approximately 40% for the cationic polymer method, 20% for the calcium phosphate precipitation technique, and less than 5% for the standard electroporation technique (Figure 2B and Figure 3B).

**Figure 4:**
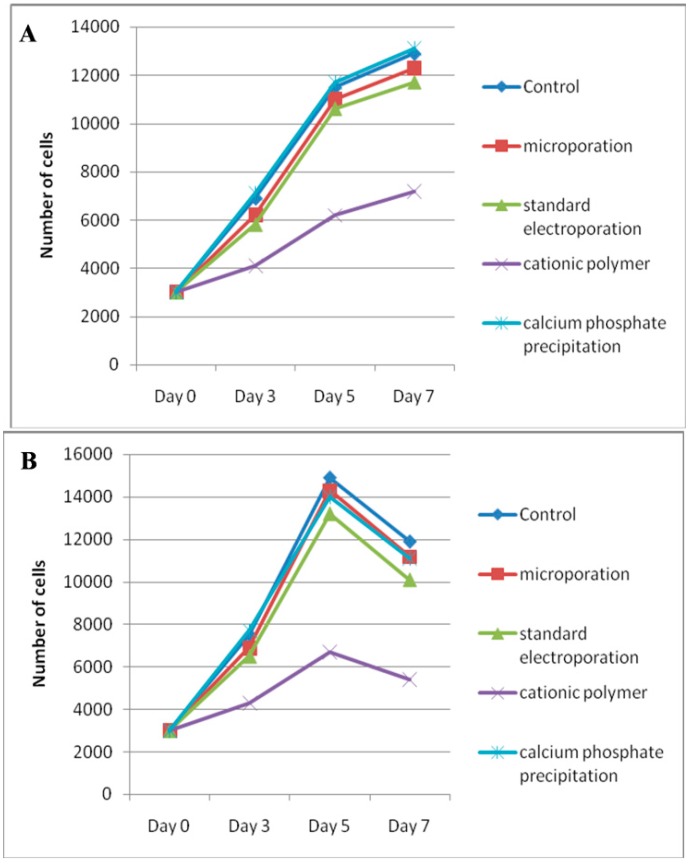
Post-transfection proliferative potential of hAD-MSCs and fibroblasts. Analysis of (**A**) hAD-MSC and (**B**) fibroblast proliferation capability following transfection using microporation, standard electroporation, cationic polymer, and classical calcium phosphate precipitation transfection techniques. Two days post-transfection, the cells were trypsinized and seeded at 3 × 10^3^ cells/well in 96-well plates. The Trypan blue exclusion assay was used to determine total cell number in the hAD-MSC and fibroblast cultures at days 3, 5, and 7 after plating. hAD-MSCs and fibroblasts transfected using the microporation, standard electroporation, and classical calcium phosphate precipitation techniques exhibited a greater proliferation potential comparable to non-transfected cells. However, transfection with the cationic polymer repressed the hAD-MSCs and fibroblasts proliferation.

To determine whether hAD-MSCs can maintain their stem cell phenotype following transfection, we considered several parameters: It was important to demonstrate that the proliferation capability of the cells was not affected and that the cells retained their ability to undergo multilineage differentiation. The proliferation capability of the cells was evaluated by comparing their relative growth capacity with that of nontransfected cells. Two days after transfection, the proliferation assay was performed with hAD-MSCs and fibroblasts grown at 37 °C and 5% CO_2_ in their specific growth medium for a seven-day period. Both nontransfected hAD-MSCs and hAD-MSCs transfected using the microporation, electroporation, and calcium phosphate precipitation transfection techniques grew in a similar fashion in a time-dependent manner (Figure 4A). However, transfection with the cationic polymer repressed the hAD-MSC proliferation rate. Likewise, the *in vitro* culture of fibroblasts appeared to be unaffected by transfection using the microporation, electroporation, and calcium phosphate precipitation strategies, but the cationic polymer transfected cells exhibited lower cell growth (Figure 4B).

**Figure 5:**
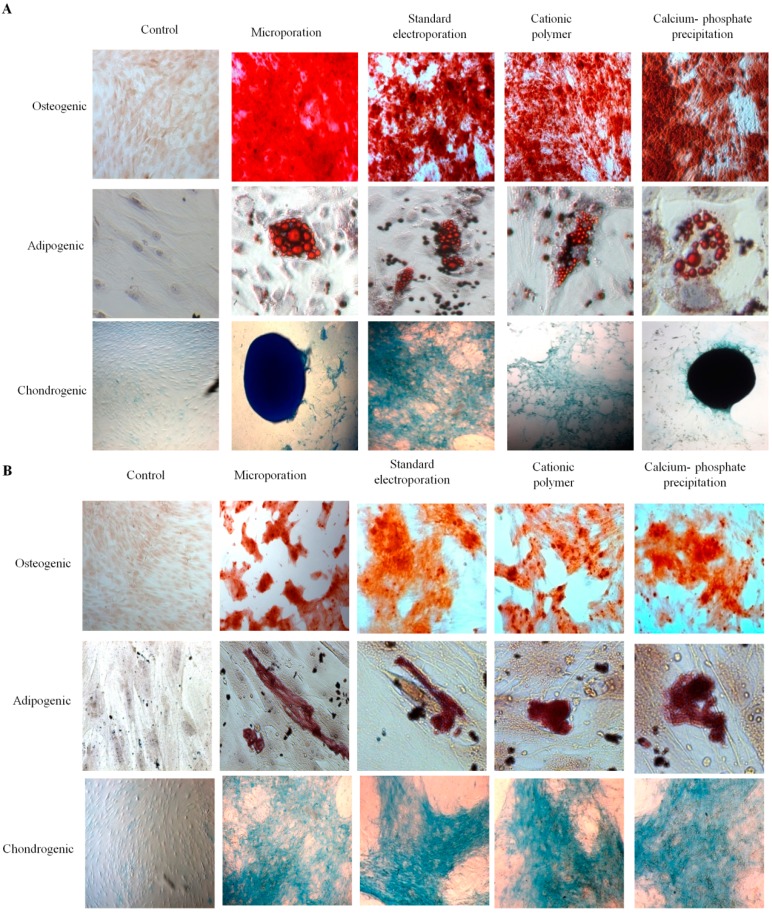
Differentiation potential of hAD-MSCs and fibroblasts following transfection using four independent techniques. Osteogenic, adipogenic, and chondrogenic differentiation of (**A**) hAD-MSCs and (**B**) fibroblasts post-transfection using the microporation, standard electroporation, cationic polymer, and classical calcium phosphate precipitation transfection techniques. After 21 days, osteogenic cultures were stained using alizarin red. Adipogenic cultures were analyzed for lipid accumulation after 14 days of differentiation using inverted microscopy. Thereafter they were fixed and stained for triglycerides with Oil-Red-O. After 21 days, chondrogenic cultures were stained for spheroid formation using Alcian blue. (Magnification: (**A**) adipogenic 400×, osteogenic 40× and chondrogenic 40×; (**B**) adipogenic 400×, osteogenic 100× and chondrogenic 100×).

Another important feature of MSCs is their ability to undergo multilineage differentiation. Thus, we induced the transfected hAD-MSC cultures to form adipogenic, osteogenic, and chondrogenic cells. Because the cationic polymer transfection reduced the proliferation rate of hAD-MSCs, all cultures were seeded at high density (6 × 10^4^ cells/well). Two days after seeding, confluent cultures were changed to osteogenic, adipogenic, chondrogenic, or control media. Osteogenic cultures were then stained after 21 days. Notably, we successfully observed apparent Alizarin red stained-cells in nontransfected control cultures and in cells transfected using all four techniques. Adipogenic differentiation was assessed after 14 days in adipogenic medium using a combination of phase contrast microscopy and Oil-Red-O staining for triglycerides. The four transfection techniques did not influence the ability of hAD-MSCs to differentiate into adipocytes based on the visible accumulation of lipid droplets and the level of Oil-Red-O staining (Figure 5A). Likewise, the chondrogenic cultures were stained for cartilage formation after culture for 21 days in chondrogenic medium. The formation of spheroids and cartilage was observed in cells transfected using all four techniques, and the results were comparable to those of the nontransfected control cultures (Figure 5A). Fibroblast cells were also able to differentiate into the three major lineages following transfection with all four techniques, and the results were comparable to those of the nontransfected fibroblast cells (Figure 5B).

## 3. Discussions

Stem cell-based therapy offers great potential for the treatment of genetic disorders. Because stem cells have been reported to restore function of damaged tissue in various preclinical disease models, combining stem cell therapy with gene therapy might provide additive benefits [1]. For example, genetically modified MSCs containing the brain-derived neurotrophic factor gene were shown to effectively promote axonal regeneration in the transected spinal cord in adult rats [12]. Mei *et al.* (2007) demonstrated that MSCs engineered to overexpress vasculoprotective factor ANGPT-1 were significantly more effective than MSCs alone in a murine model of acute lung injury (ALI) [13]. These reports suggest a potential role for genetic manipulation of MSCs in treating clinical ALI and various other diseases. Unfortunately, *ex vivo* genetic manipulation of MSCs (and other primary cells) is challenging because they are resistant to commonly used non-viral transgene delivery methods to introduce exogenous DNA or RNA (*i.e.*, liposome-based, cationic polymer, electroporation, and calcium phosphate precipitation techniques) [4,14,15].

The results of the present study demonstrate the difficulty in transfecting hAD-MSCs with plasmid regulating eGFP using classic non-viral methods (*i.e.*, the calcium phosphate precipitation, cationic polymer, and standard electroporation techniques). These results agree with those presented by Zaragosi and coworkers, who reported that fewer than 5% of hAD-MSCs were eGFP positive when calcium phosphate precipitation and lipofectants (*i.e.*, Lipofectamine 2000 and FuGENE transfection reagents) were used [15]. Aluigi *et al.* (2006) reported that the percentage of GFP-expressing human bone marrow-derived MSCs (BM-MSCs) transfected with two different lipid-based non-viral systems was 4.4% for FuGENE6 and 6.8% for DOTAP Liposomal Transfection Reagent [16]. In addition, our data showed that transfection with the cationic polymer slightly repressed hAD-MSC and fibroblast proliferation. A possible explanation for this observation might be that the cationic polymer formulation has a toxic effect. Overall, these observations suggest that classical transfection methods are generally less efficient than viral-based techniques [16]. In addition, the classic techniques cannot be used with slowly proliferating cells, and they result in high mortality due to toxicity [15].

Safety and efficiency are major concerns for stem cell therapy involving the transfer of therapeutic transgenes. Although the use of viral vectors can result in more stable transgene expression and higher transfection efficiencies compared to non-viral methods, a major concern is the associated risk of inducing toxicity in the recipient body and triggering the immune and inflammatory responses [3,17,18,19]. Furthermore, virus-based experiments are difficult to set up because of rigid requirements for upholding specific safety conditions, especially when using human cells [20]. In contrast, non-viral methods typically have a transfection efficiency of 20%–25%, which is less than 80%–90% of viral vectors. However, non-viral methods offer several advantages, including lower manufacturing costs and the lack of immunogenic responses following repeated administration [6].

Electroporation is a non-viral transfection technique that uses a short high voltage pulse to electropermeabilize the cell membrane, which enables the DNA to penetrate the cell membrane; this method can lead to high transfection efficiencies but also high cell mortality [16,21]. Helledie *et al.* (2008) had optimized the parameters of the electrotransfer of human MSCs (hMSCs), using exponential decaying pulses, which resulted in up to 90% stable transfection efficiency but with about 50% cell viability suggested that this technique is rather harsh and caused a high rate of cell mortality, especially in MSCs, thus undermined the ability to transfer genes into these cells [22,23]. Excessive cell death may be due to the large surface area of the electrode. To address this issue, Invitrogen-Life Technologies, Grand Island, NY, USA, developed an advanced electroporation system called the Neon™ Transfection System or, in short, microporation [4,11]. The goal of developing this microporation system was to enhance transfection efficacy and MSC viability and to overcome some of the limitations of the standard electroporation and chemical-based transfection techniques. In microporation, cells are contained within a capillary style chamber as opposed to the standard electroporation cuvette. This makes it possible to decrease the electrode surface area and minimize pH variation, temperature variation, and metal ion release, all of which contribute to poor cell survival during traditional electroporation [4]. Phillips *et al.* (2007) reported that the transfection efficiency of BM-MSCs using the microporation technique was up to 40% and cell viability was up to 85% [20]. In the current study, we found that a plasmid encoding the transgene, could be routinely transfected into hAD-MSCs via microporation at efficiencies of about 50% with a concurrent 78% viability. Furthermore, unlike traditional electroporation strategies, this microporation technique results in minimal death of hAD-MSCs [4,6,14,24]. Therefore, the technique should allow for the production of a sufficient quantity of transfected hAD-MSCs for cell therapy without the need for extensive cell culturing.

The evaluation of several parameters when identifying optimum transfection condition with electroporation including, pulse magnitude, pulse duration, pulse frequency, plasmid concentration and the cell concentration. Furthermore, researchers must consider the cell type, the desired outcome (*i.e.*, relative importance of transfection efficiency and viability) and electroporation system to be used. Hence the number of potential variations is large and evaluating every variation is not practically feasible. To narrow the scope, we identified the pulse magnitude as the most important parameter as minor variations in pulse magnitude can dramatically impact the cell survival. Our approach was to identify the optimum conditions for transfection of hAD-MSCs and to test variations in pulse magnitude (1000 to 1600 V)

As is true for most plasmid-based transfection methods, we found that microporation induced only transient transgene expression in hAD-MSCs [24]. We detected maximum transient transgene expression at day two post-microporation. Thus, this approach would be useful for short-term therapies, as the expression of eGFP in microporated hAD-MSCs gradually decreased over time due to dilution or to decreases in the number of plasmid copies in the cells. However, even after three weeks in culture, expression of eGFP in microporated hAD-MSCs could still be detected. As previously shown by others using human umbilical cord blood-derived MSCs transfected with the plasmid encoding for the brain-derived neurotrophic factor gene, expression of the transgene remained fairly constant for the first two weeks both *in vitro* and *in vivo* [4]. In contrast, Sitton *et al.* (2006) reported that the plasmid copy number in the ovary cells of Chinese hamsters that were transfected using the electroporation method decreased exponentially in seven days [25]. Viral delivery systems are frequently used for long-term expression of a gene of interest, but for many non-inherited diseases it may be desirable to express the therapeutic gene transiently. Because the transfected gene was only expressed for a short period of time (~21 days) in this study, microporation is an ideal gene delivery technique for this sort of therapeutic approach. For example, this approach could be used to generate transient expression of anti-inflammatory genes to suppress acute inflammation or transient expression of homing chemokines to move MSCs toward the site of injury [26].

Given the expression of ANGPT-1 convey the anti-inflammatory, antipermeability, and endothelial-protective characteristics in the cells, MSCs transfected with this gene can effectively promote lung repair and also protect the lung from chronic or acute lung injury. Mei *et al.* (2007) reported that treatment with MSCs alone significantly reduced Lipopolysaccharide (LPS)-induced acute pulmonary inflammation in mice, whereas administration of MSCs transfected with the human ANGPT1 plasmid resulted in further improvement by completely reversing LPS-induced permeability in the lung of the ALI model [13]. Thus, hAD-MSCs transfected with the *ANGPT-1* gene via microporation are potential candidates for the treatment of lung injuries, and this technique could be implemented to produce an adequate quantity of transfected hAD-MSCs for cell therapy.

The findings from this study provide information that can be used to select a method to transfect the gene of interest into human MSCs. The four transfection techniques tested in this study did not influence the ability of hAD-MSC to differentiate into the three different mesodermal lineages. The proliferation capability of these cells was also independent of the transfection method used, with the exception of the cationic polymer technique, which slightly repressed the proliferation of hAD-MSCs and fibroblast cells. However, we found that the microporation technique had the great potential to enhance the transfection efficiency without losing the cells’ multipotency and proliferative characteristics. Maintenance of these properties in modified stem cells is critical for improving their therapeutic potential. Otherwise, stem cells may not be effective in a combination therapy using stem cells and therapeutic genes [4].

We used fibroblast cells as a control non-stem cell in this study because of their similarity to MSCs. They share some functional similarities, including surface markers and some differentiation ability [27]. Though our results demonstrated that a large number of fibroblast cells were positive for eGFP following transfection using the microporation technique as compared to the microporated hAD-MSCs. Interestingly, we found that fibroblast cells transfected using the calcium phosphate precipitation method had 20% transfection efficiency. This was unexpected, as it is usually difficult to transfect primary cells using this classical method. In addition, the capability of fibroblast cells to proliferate and differentiate into the three major lineages was independent of the transfection technique used in this study. Transfection of these two primary cells, hAD-MSCs and the fibroblast cells showed that the microporation technique was more effective than the two chemical reagents tested as well the standard electroporation method.

## 4. Materials and Methods

This study was carried out in strict accordance with a recommendation from the university guidelines. The protocol was approved by the animal ethics committee of Universiti Sains Malaysia (USM/Animal Ethics Approval/2012/(80) (383)).

### 4.1. Plasmid

A precision LentiORF expression vector, pLOC/ANGPT1/eGFP (9.9 kb) was purchased from Thermo Scientific, Waltham, MA, USA. In pLOC/ANGPT1/EGFP, the enhanced green fluorescent protein (eGFP) and Angiopoietin-1 (ANGPT1) expression are driven by a cytomegalovirus (CMV) promoter. Its unique design expressed the ORF, the transgene (*ANGPT1*), the fluorescent reporter and the selection marker from one promoter, providing a visual indicator for ORF expression and a mechanism for selecting transfected cells. This plasmid was purified using an endotoxin-free plasmid DNA purification kit protocol (Macharey-Nagel, Duren, Germany). The concentration of purified pDNA solutions was assayed by spectrophotometry at 260 nm (Nanodrop, Thermo Scientific, Waltham, MA, USA).

### 4.2. Cell Cultures

hAD-MSCs (American Type Culture Collection, Manassas, VA, USA) were at passage zero upon received and cultured in growth medium, which consisted of low glucose Dulbecco’s Modified Eagle’s Medium (DMEM, 1000 mg/L) supplemented with 10% fetal bovine serum (FBS) and 2 mM l-glutamine containing 1% penicillin/streptomycin (all from Gibco-Life Technologies, Grand Island, NY, USA). Fibroblasts, which were used as control cells, were obtained from the skin of an adult New Zealand white rabbit. They were cultured until 80% confluent in the growth medium (high glucose DMEM (4500 mg/L) supplemented with 10% FBS and 1% penicillin/streptomycin). Both hAD-MSCs and fibroblasts were grown at 37 °C in humidified air containing 5% CO_2_ and were subcultured every 4–5 days.

For the transfection assay, low passage hAD-MSCs (passage 5) and fibroblasts (passage 5) were expanded and transfected with the pLOC/ANGPT1/eGFP. Transfection assays were performed using four independent techniques: microporation, standard electroporation, cationic polymer, and calcium phosphate precipitation.

### 4.3. Immunophenotyping of Human Adipose-Derived MSCs (hAD-MSCs)

To analyze the cell surface expression of MSCs marker protein in hAD-MSCs, cells were labeled with the following anti-human antibodies: CD44-FITC, CD73-PE, CD90-PE, CD105-PE, CD271-FITC, CD34-FITC, CD15-FITC, and CD45-PE (BD Biosciences, Palo Alto, CA, USA). One million cells were measured using FACSCalibur flow cytometer (BD Biosciences) and the results were analyzed with CellQuest software (BD Biosciences).

### 4.4. Calcium Phosphate Precipitation

For the calcium phosphate precipitation transfection method, approximately 4 × 10^4^ hAD-MSCs or fibroblasts were seeded in 6-well plates containing antibiotic-free growth medium 24 h prior to transfection. Three hours prior to transfection, the medium was replaced with 2 mL fresh growth medium. In a sterile polystyrene tube, 2, 4, 6, 8, and 10 µg of plasmid DNA were added to 0.1 mL of 0.25 M calcium chloride (CaCl_2_). Subsequently, 0.1 mL of 2× BES-buffered saline (BBS) was added to the DNA solution and mixed well. The DNA/CaCl_2_/BBS mixture then was added to the cells, and the plates were gently rocked to ensure even distribution. The cells were incubated at 37 °C in a 5% CO_2_ incubator for 15–24 h. The cells were washed twice with PBS and changed to fresh growth medium. The cells then were incubated for an additional 24 h at 37 °C in a 5% CO_2_ incubator. The transfection complexes, consisting of different transfection reagent/DNA ratio (*v*/*w*) of CaCl_2_/BBS/DNA were: 1/1/2, 1/1/4, 1/1/6, 1/1/8, and 1/1/10 respectively.

### 4.5. Cationic Polymer

For transfection using the cationic polymer technique, both cell types were transfected with TurboFect Transfection Reagent (Thermo Scientific) according to the manufacturer’s protocol. Briefly, the cells were seeded at 4 × 10^4^ cells/well in 6-well plates containing antibiotic-free growth medium 24 h prior to transfection. The plasmid DNA was diluted in the growth medium. The volume of DNA should be 1/10 of the volume of culture medium. Six microliter of TurboFect Transfection Reagent were added to the diluted DNA, mixed thoroughly and incubated at room temperature for 15 min according to the manufacturer’s instructions. The reagent/DNA mixture was added to the medium and the cells were incubated at 37 °C in a 5% CO_2_ incubator. The *v*/*w* ratios of Turbofect/DNA were: 6/2, 6/4, 6/6, 6/8, and 6/10 respectively.

### 4.6. Microporation

For the microporation technique, the Neon™ Transfection System (Invitrogen-Life Technologies, Grand Island, NY, USA) was used to transfect both hAD-MSCs and fibroblast cells. Cells were prepared per the manufacturer protocol. Briefly, microporation was performed with 2 μg of DNA plasmid per 2.0 × 10^5^ cells per transfection. Cells were transfected with pLOC/ANGPT1/eGFP reporter plasmid and plated in 24-well plates with fresh medium. A control cellular fraction was plated at the same density without microporation. Initial optimization was performed following the manufacturer’s protocol (Pulse voltage: 1400 V; pulse width: 10 ms; pulse number: 1; and 10 μL tip type). To further refine the microporation protocol, additional conditions were evaluated, with a single pulse applied in the range of 1000–1600 V at 100 V increments for duration of 20 ms for both sells. There were seven different pulsing programs tested for hAD-MSCs: program 1 (P1), 1000 V, 20 ms, one pulse; (P2) 1100 V, 20 ms, one pulse; (P3) 1200 V, 20 ms, one pulse; (P4), 1300 V, 20 ms, one pulse; (P5) 1400 V, 20 ms, one pulse; (P6) 1500 V, 20 ms, one pulse and (P7) 1600 V, 20 ms, one pulse. After microporation, the cells were transferred to a tissue culture plate, where they were incubated at 37 °C in a humidified CO_2_ incubator.

### 4.7. Standard Electroporation

For standard electroporation, cells were resuspended in a serum- and antibiotic-free DMEM at 1 × 10^6^ cells/mL. A total of 200 μL of cells were mixed with 10 μg DNA transferred to a 4 mm electroporation cuvette (BioRad, Hercules, CA, USA), and electroporated using Gene Pulser Xcell electroporation system (BioRad). The electroporation was done with pulse magnitude was varied from 80 to 120 V and the pulse duration (ms) and frequency (pulse number) were held constant at 20 ms and 1 per transfection, respectively. After electroporation, the cells were incubated for 5 min at room temperature and transferred to the tissue culture dishes containing the growth medium. After 24 h, the medium was changed to remove debris and dead cells.

### 4.8. Assessment of Transfection and Detection of Enhanced Green Fluorescent Protein (eGFP) Expression

The eGFP expression of cells that were transfected using the different transfection strategies was quantitatively determined 48 h post-transfection using phase contrast and fluorescence microscopy. They were calculated as a percent of eGFP^+^ cells relative to the total number of cells per field (*n* > 4 field per condition; fields were selected randomly based on the quadrant drawn on the well). Cell count were performed for all conditions and the viability was determined by Countess™ automated cells counter (Invitrogen) as a percentage of the total number of transfected cells per well relative to the total number of cells per well in control cultures. Expression was measured every 2 days for 3 weeks, and then the cells were seeded for the proliferation and differentiation assays.

### 4.9. Proliferation Assay

For the proliferation assays, cells were seeded at 3 × 10^3^ cells/cm^2^ in 96-well plates and cultured for 7 days in the growth medium. Medium was refreshed every 3 days. At days 3, 5, and 7, the number of viable cells was determined using the Trypan blue exclusion assay.

### 4.10. Differentiation Assay

The hAD-MSCs and fibroblast cells were induced to differentiate into three lineages: adipogenic, osteogenics, and chondrogenic cells. For adipogenic differentiation, approximately 6 × 10^4^ of the hAD-MSC cells were seeded in a 24-well tissue plate containing normal growth medium (low glucose DMEM supplemented with 10% FBS and 1% penicillin streptomycin). The cells were prepared in triplicate and cultured to reach 100% confluence (24–48 h). Once the cells had reached confluence, they were induced to differentiate into adipogenic cells by replacing the culture medium with adipogenic differentiation medium (Promo Cell, Heideberg, Germany). The remaining wells containing normal growth medium served as the control. The cells were incubated for 14 days and the medium was changed every third day. After 14 days, cells were stained with Oil-Red-O (Sigma, St. Louise, MO, USA). For the osteogenic differentiation assay, the cells were seeded at 6.0 × 10^4^ cells/well in a 24-well tissue culture plate containing the normal growth medium. The cells were cultured to reach 100% confluence, at which time the medium was changed to osteogenic differentiation medium (Promo Cell). The medium was changed every third day. The remaining wells containing normal growth medium served as the control. After 21 days, cells were stained with Alizarin red (Sigma). For the chondrogenic differentiation assay, the cells were seeded at 1 × 10^5^ cells/well in a 96-well tissue culture plate and cultured to reach confluence. The cells were induced to differentiate into chondrogenic cells by replacing the growth medium with chondrogenic differentiation medium (Promo Cell). They were cultured for 21 days, and the medium was changed every third day. The remaining wells containing normal growth medium served as the control. After 21 days, the formation of cartilage was detected using Alcian blue (Sigma) staining. The differentiated cells and negative controls were analyzed using the inverted microscope.

## 5. Conclusions

In conclusion, we have demonstrated that the microporation technique is an effective method for gene transfection into hAD-MSCs, especially for applications requiring only low and transient expression of proteins. The stem cell plasticity of hAD-MSCs was maintained after microporation. With a transfection efficiency of up to 50% and no negative effect on the cell survival, proliferation and differentiation capabilities of MSCs, the microporation technique may prove beneficial for future application in *in vivo* stem cell-based gene therapy.

## Supplementary Files

Supplementary File 1
