# Supplementary material for: A Comparative Study of Non-Viral Gene Delivery Techniques to Human Adipose-Derived Mesenchymal Stem Cell
Source: Int J Mol Sci. 2014 Aug 26;15(9):15044–60. doi: 10.3390/ijms150915044 (PMC4200830; doi:10.3390/ijms150915044)
Supplement: Supplementary File 1 [file ijms-15-15044-s001.pdf]

Supplementary Information

Figure S1. Optimization of hAD-MSCs and fibroblasts transfection by microporation technique. hAD-MSCs and fibroblasts were transfected with 2 μg of pLOC/ANGPT1/eGFP using a microporator. (**A**) After 48 h, the expression of eGFP was analyzed using fluorescence microscopy. Cells were microporated using seven different pulse conditions: (P1) 1000 V, 20 ms, one pulse; (P2) 1100 V, 20 ms, one pulse; (P3) 1200 V, 20 ms, one pulse; (P4), 1300 V, 20 ms, one pulse; (P5) 1400 V, 20 ms, one pulse; (P6) 1500 V, 20 ms, one pulse and (P7) 1600 V, 20 ms, one pulse. Scale bar = 100 μm; (**B**) Cell viability were defined as a percentage of microporated cell per well relative to the total number of cells per well in control wells and the relative percentage was plotted as a function of pulse magnitude. The transfection score was maximized at a pulse magnitude of 1500 V for hAD-MSCs and 1400 V for fibroblasts and hence were identified as the optimum condition for hAD-MSCs and fibroblasts transfection.

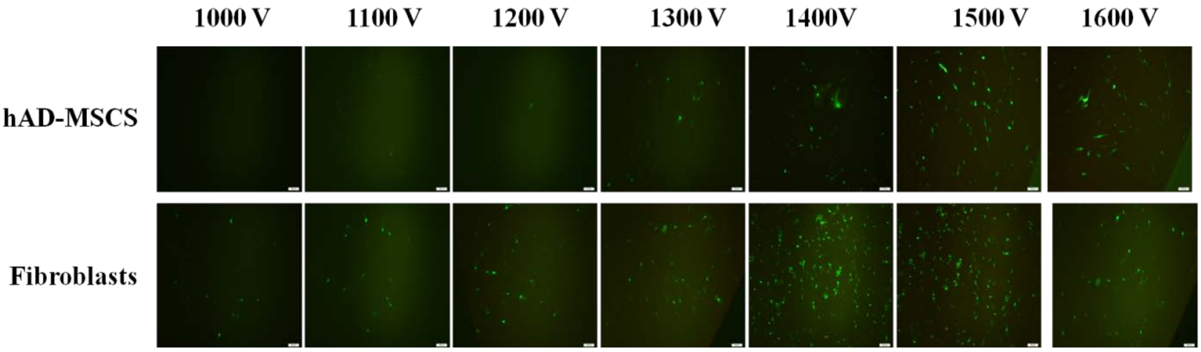

(**A**)

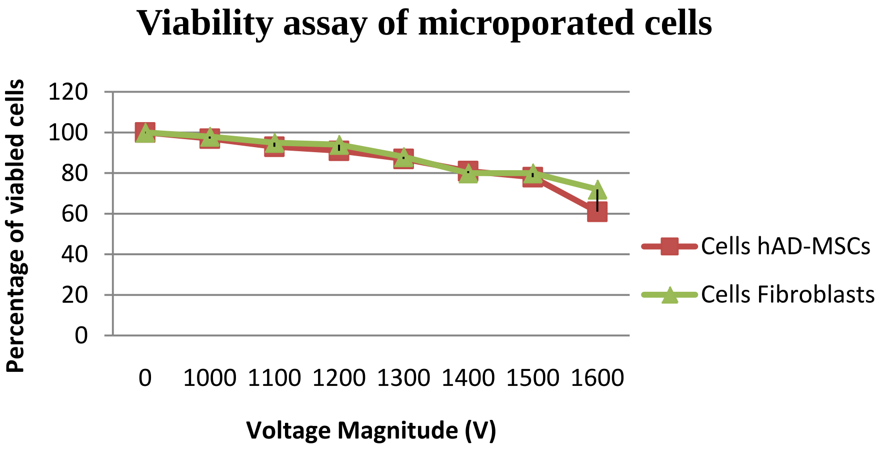

(**B**)

Figure S2. Determination of transfection efficiency by transfection reagents of different concentration of plasmid DNA into hAD-MSCs and fibroblasts. Cells were transfected with pLOC/ANGPT1/eGFP plasmid over a range of reagent/DNA ratios (*v*/*w*) of (**A**) cationic polymer and (**B**) calcium phosphate precipitation techiques. The *v*/*w* ratios of Turbofect/DNA were: 6/2, 6/4, 6/6, 6/8 and 6/10. While the *v*/*w* ratios of $CaCl_2$/BBS/DNA were: 1/1/2, 1/1/4, 1/1/6, 1/1/8 and 1/1/10 respectively as described in Materials and Methods. The expression level of eGFP was analyzed using fluorescence microscopy; Cell viability was defined as a percentage of transfected cell per well relative to the total number of cells per well in control wells (**C**) and (**D**). For fibroblasts the highest transfection efficiency was obtained at *v*/*w* ratio of 6/10 for cationic polymer and 1/1/10 for calcium phosphate precipitation. Scale bar = 100 µm.

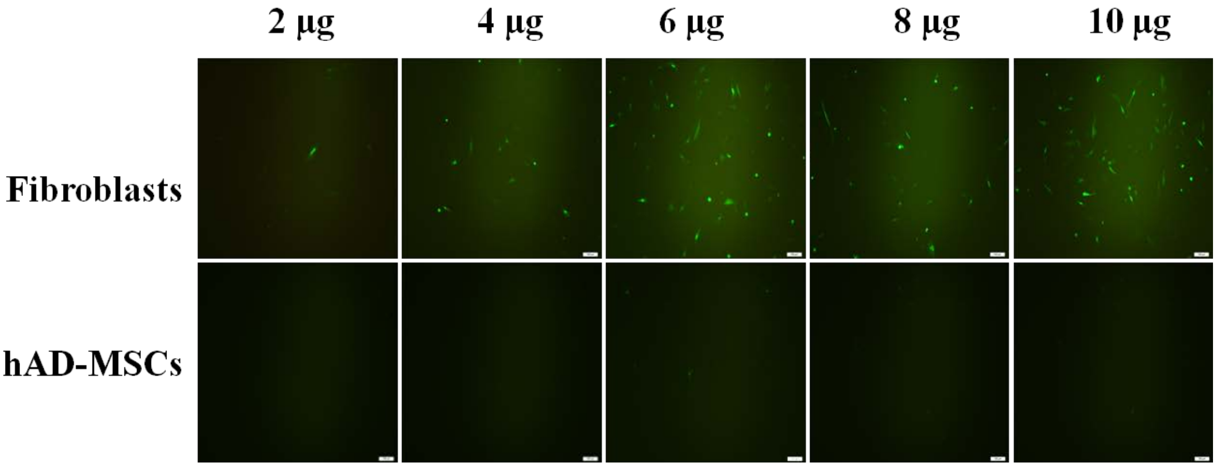

(**A**)

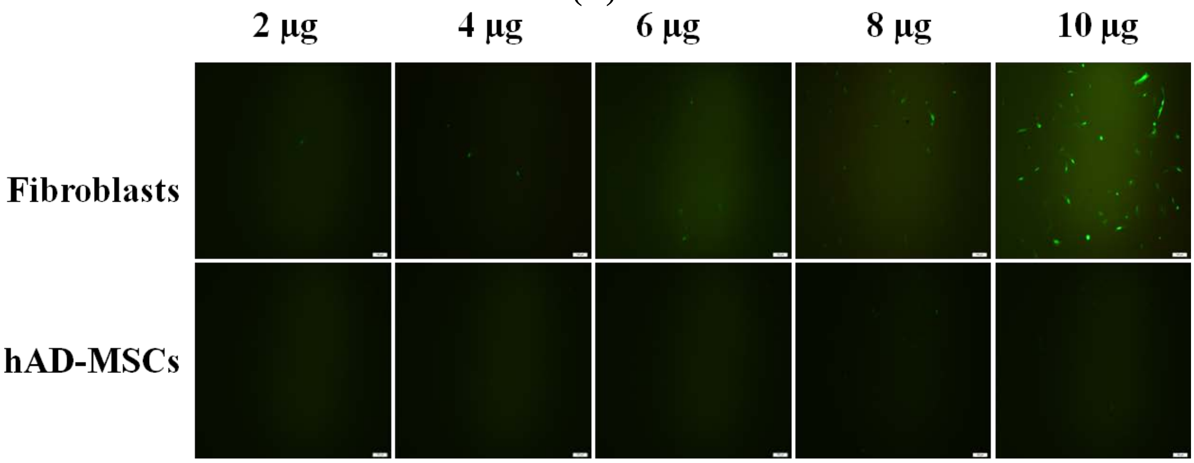

(**B**)

Figure S2. *Cont.*

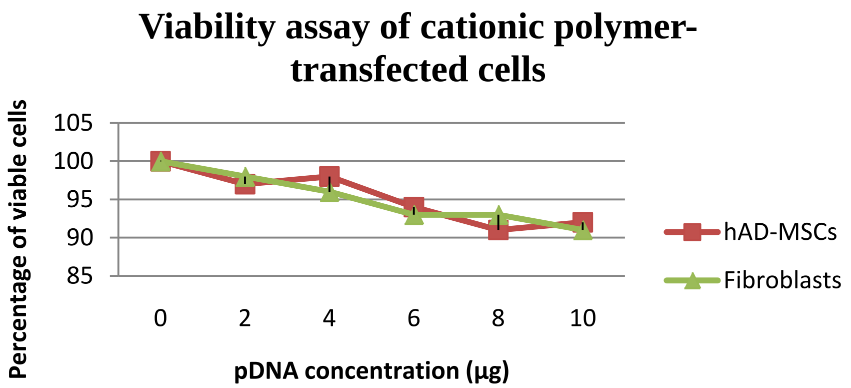

(C)

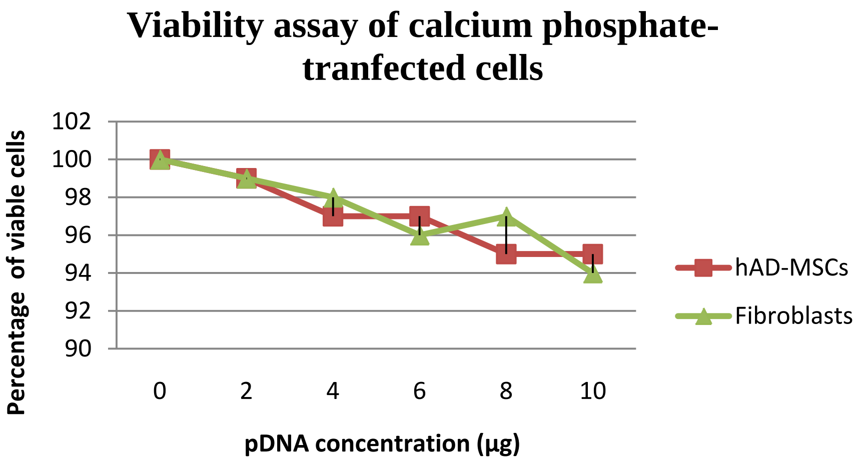

(D)

Figure S3. Optimization of hAD-MSCs and fibroblasts transfection by standard electroporation technique. hAD-MSCs and fibroblasts were transfected with 10 µg of pLOC/ANGPT1/eGFP using a Gene Pulser Xcell electroporation system. (**A**) After 48 h, the expression of eGFP was analyzed using fluorescence microscopy. Cells were electroporated using square wave mode with pulse magnitude was varied from 80–120 V and the pulse duration (ms) and frequency (pulse number) were held constant at 20 ms and 1 per transfection. Scale bar = 100 µm; (**B**) Cell viability were defined as a percentage of electroporated cell per well relative to the total number of cells per well in control wells and the relative percentage was plotted as a function of pulse magnitude**.** The transfection effieciency was maximized at a pulse magnitude of 120 V for both cells.

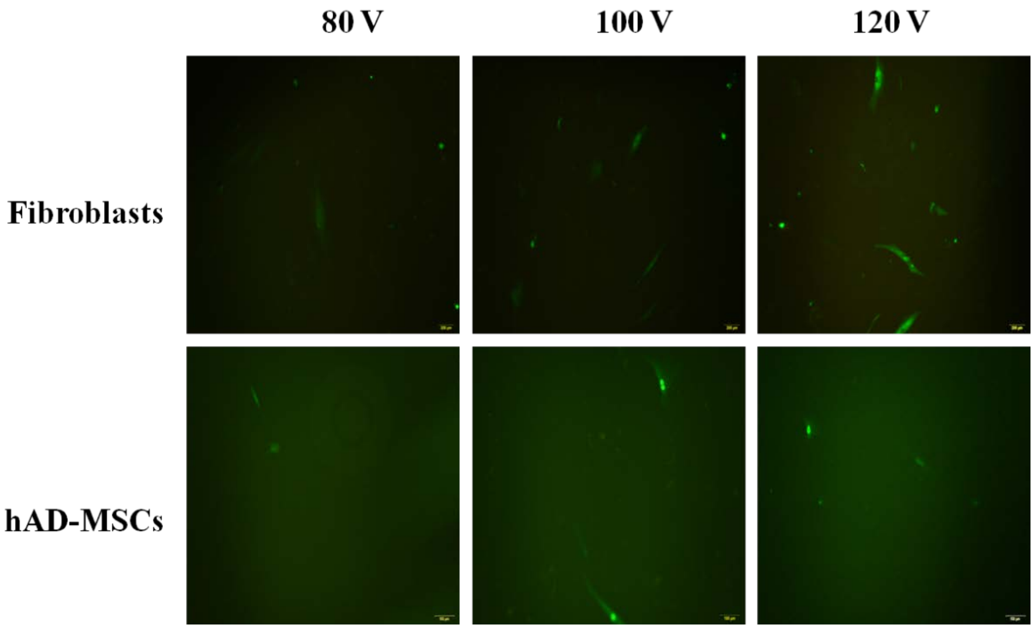

(**A**)

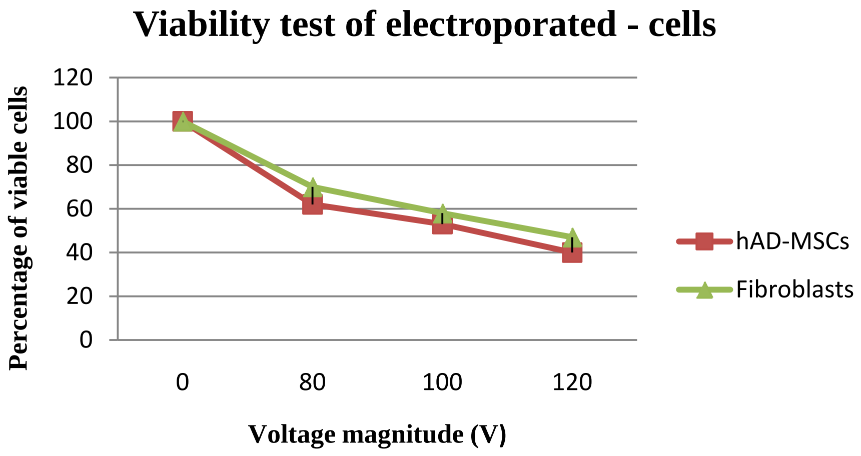

(**B**)
